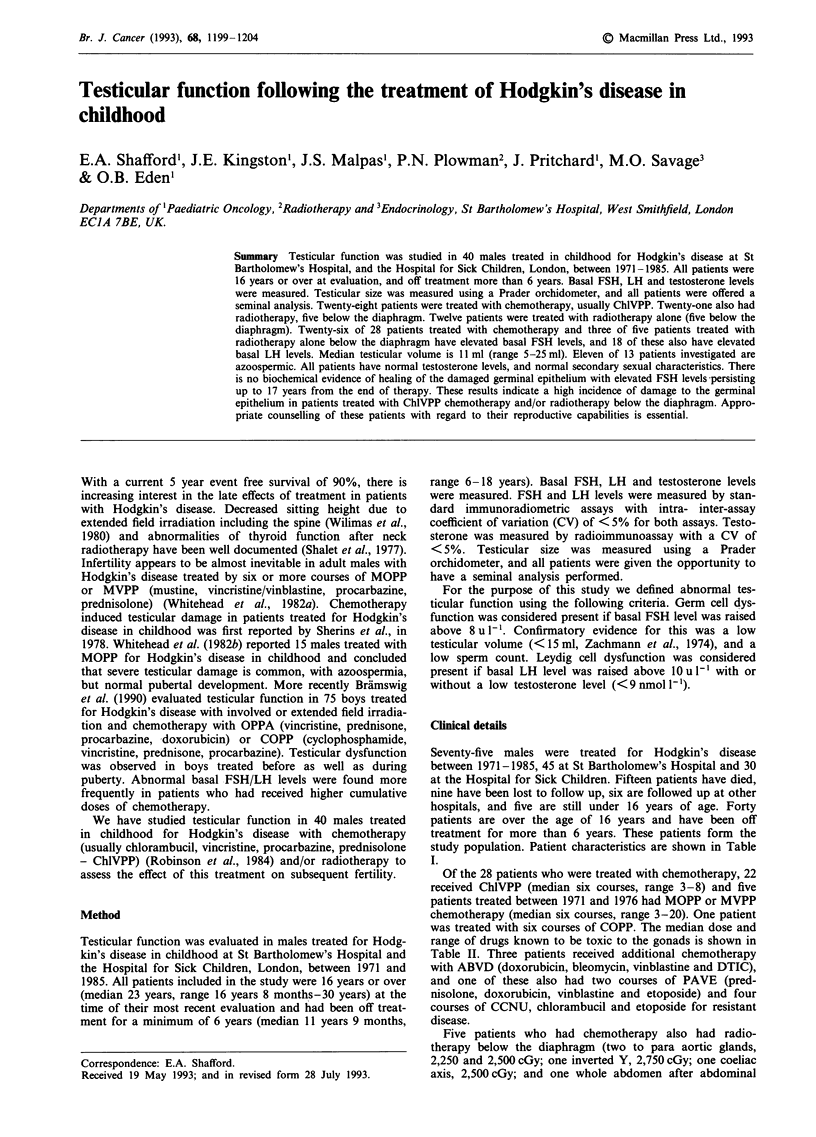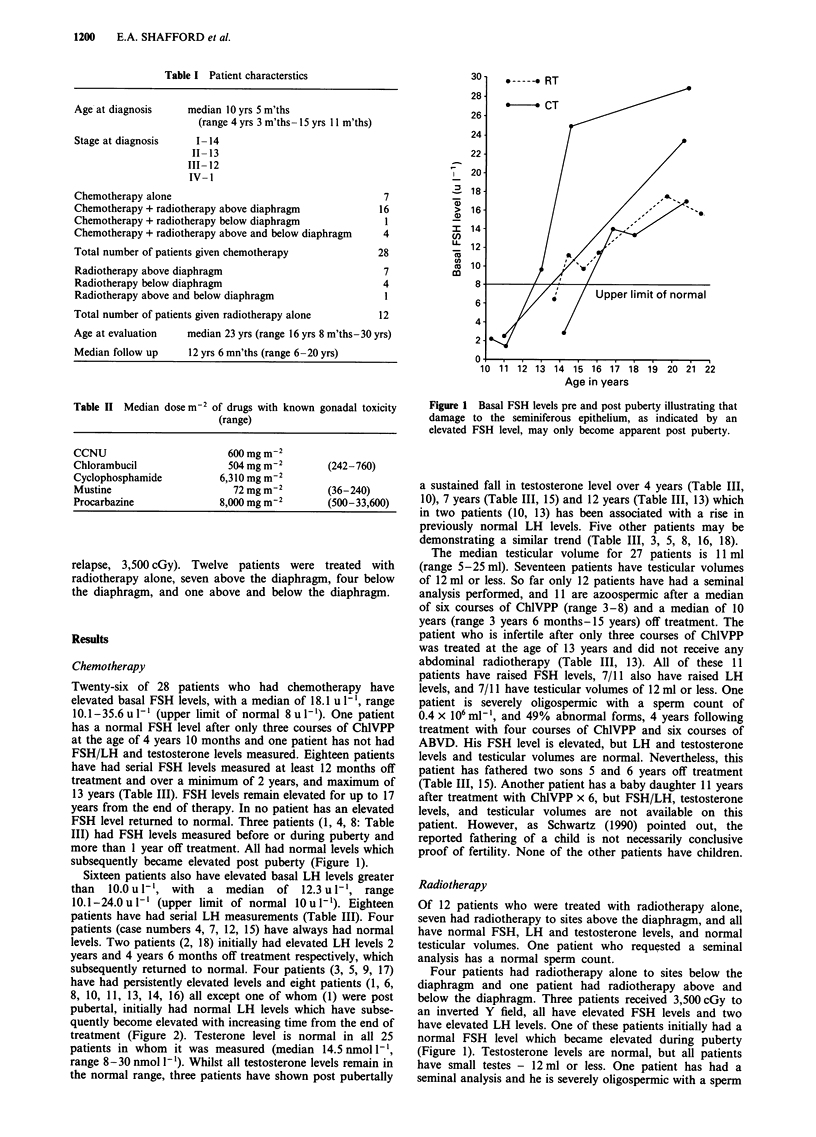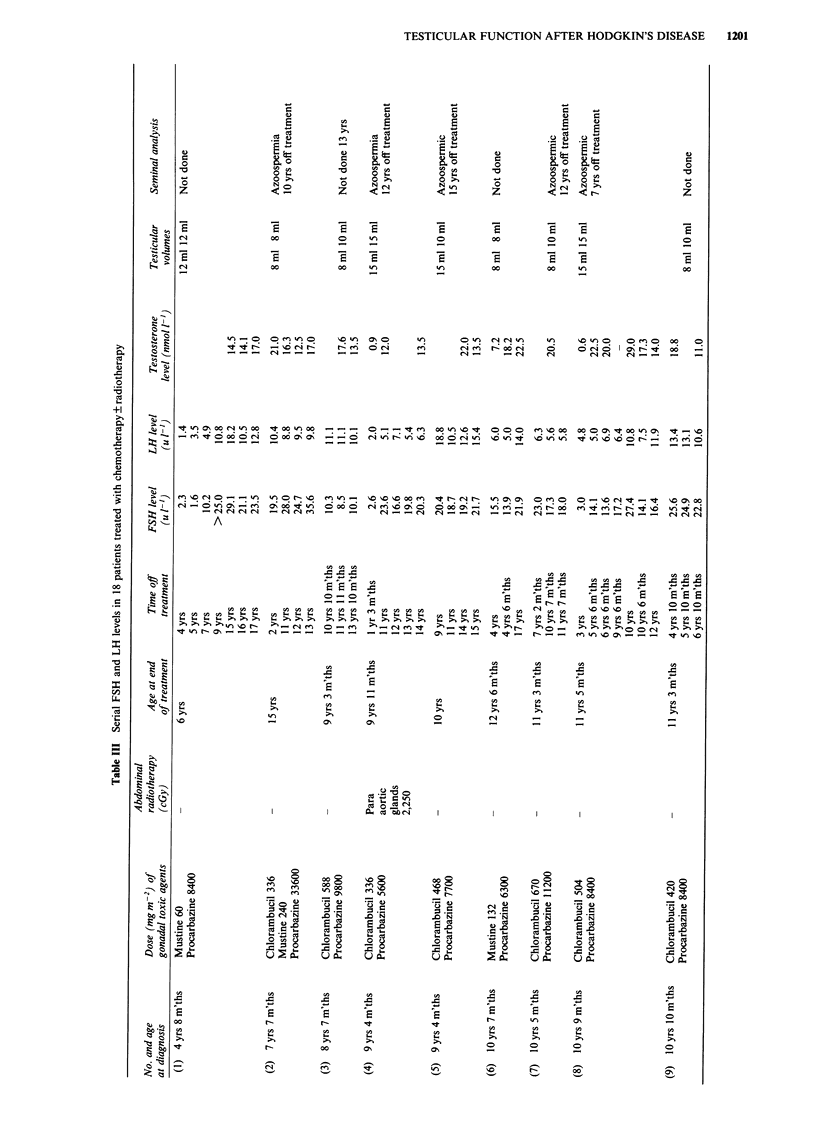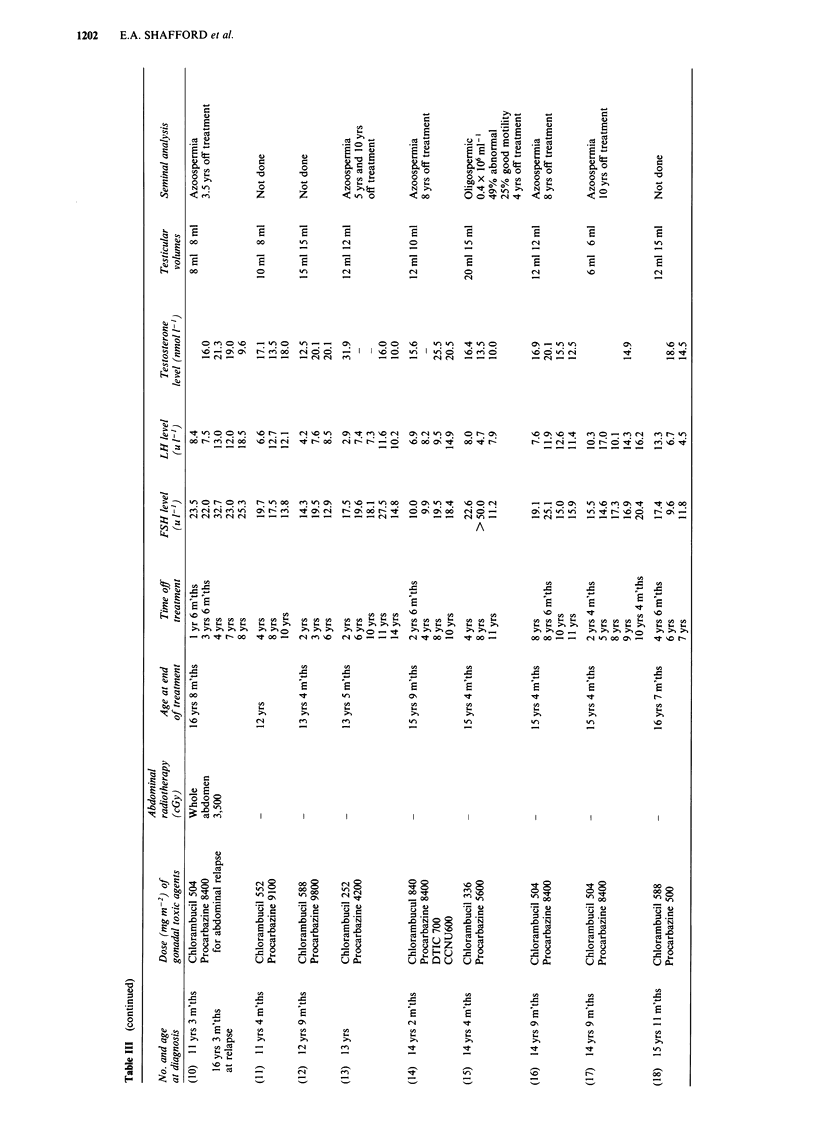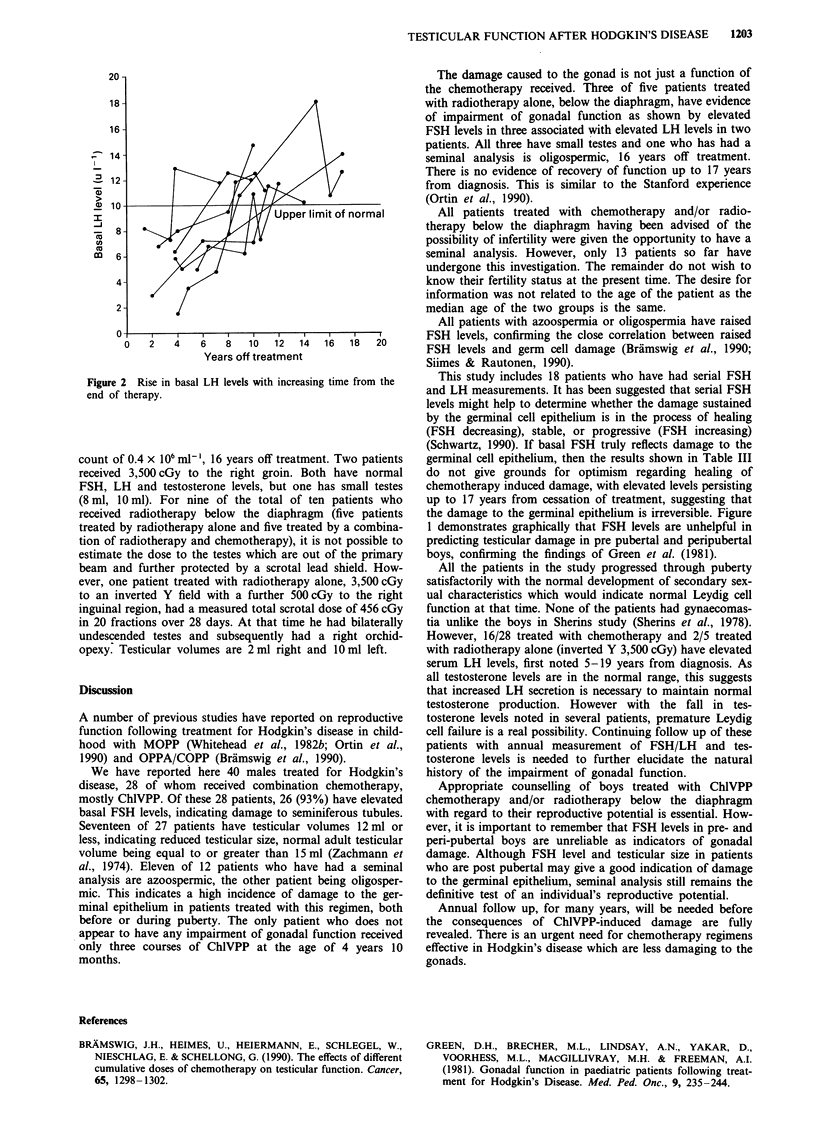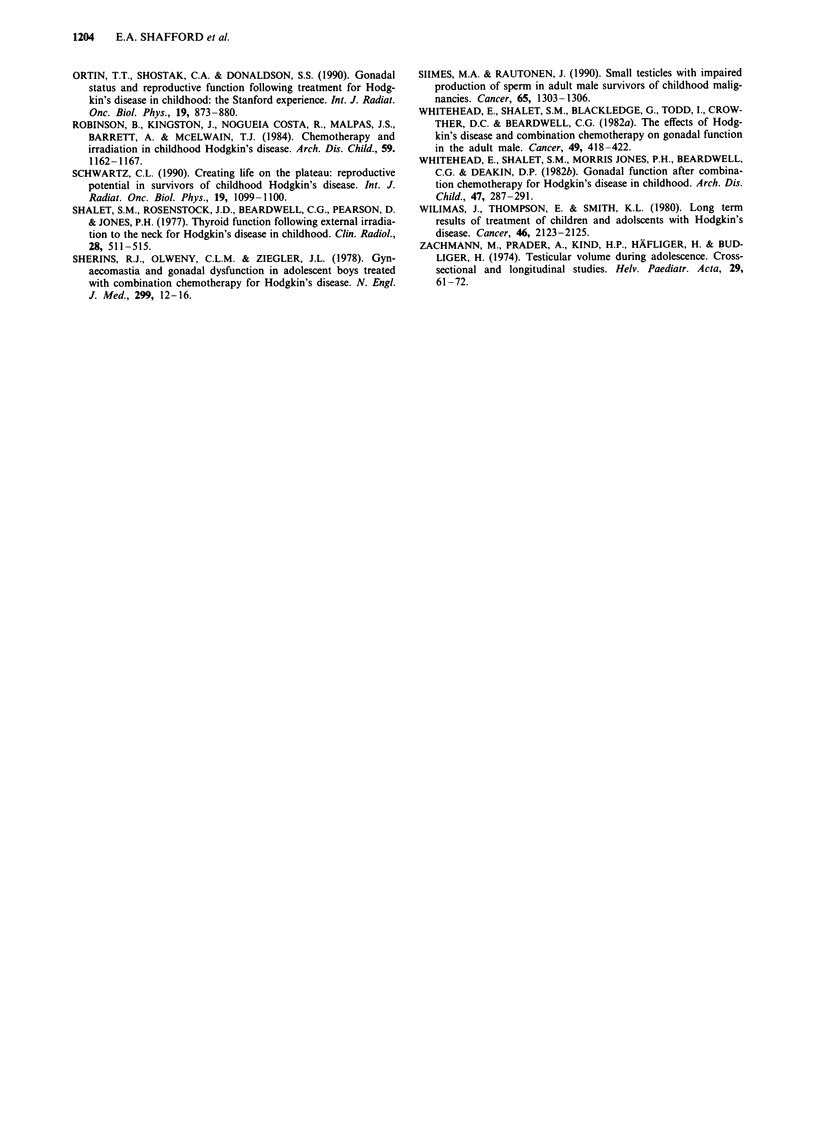# Testicular function following the treatment of Hodgkin's disease in childhood.

**DOI:** 10.1038/bjc.1993.504

**Published:** 1993-12

**Authors:** E. A. Shafford, J. E. Kingston, J. S. Malpas, P. N. Plowman, J. Pritchard, M. O. Savage, O. B. Eden

**Affiliations:** Department of Paediatric Oncology, St Bartholomew's Hospital, West Smithfield, London, UK.

## Abstract

Testicular function was studied in 40 males treated in childhood for Hodgkin's disease at St Bartholomew's Hospital, and the Hospital for Sick Children, London, between 1971-1985. All patients were 16 years or over at evaluation, and off treatment more than 6 years. Basal FSH, LH and testosterone levels were measured. Testicular size was measured using a Prader orchidometer, and all patients were offered a seminal analysis. Twenty-eight patients were treated with chemotherapy, usually ChlVPP. Twenty-one also had radiotherapy, five below the diaphragm. Twelve patients were treated with radiotherapy alone (five below the diaphragm). Twenty-six of 28 patients treated with chemotherapy and three of five patients treated with radiotherapy alone below the diaphragm have elevated basal FSH levels, and 18 of these also have elevated basal LH levels. Median testicular volume is 11 ml (range 5-25 ml). Eleven of 13 patients investigated are azoospermic. All patients have normal testosterone levels, and normal secondary sexual characteristics. There is no biochemical evidence of healing of the damaged germinal epithelium with elevated FSH levels persisting up to 17 years from the end of therapy. These results indicate a high incidence of damage to the germinal epithelium in patients treated with ChlVPP chemotherapy and/or radiotherapy below the diaphragm. Appropriate counselling of these patients with regard to their reproductive capabilities is essential.


Br. J. Cancer (1993), 68, 1199–1204 © Macmillan Press Ltd., 1993

### Testicular function following the treatment of Hodgkin's disease in childhood

E.A. Shafford[1], J.E. Kingston[1], J.S. Malpas[1], P.N. Plowman[2], J. Pritchard[1], M.O. Savage[3] & O.B. Eden[1]

*Departments of [1]Paediatric Oncology, [2]Radiotherapy and [3]Endocrinology, St Bartholomew's Hospital, West Smithfield, London EC1A 7BE, UK.*

**Summary** Testicular function was studied in 40 males treated in childhood for Hodgkin's disease at St Bartholomew's Hospital, and the Hospital for Sick Children, London, between 1971–1985. All patients were 16 years or over at evaluation, and off treatment more than 6 years. Basal FSH, LH and testosterone levels were measured. Testicular size was measured using a Prader orchidometer, and all patients were offered a seminal analysis. Twenty-eight patients were treated with chemotherapy, usually ChlVPP. Twenty-one also had radiotherapy, five below the diaphragm. Twelve patients were treated with radiotherapy alone (five below the diaphragm). Twenty-six of 28 patients treated with chemotherapy and three of five patients treated with radiotherapy alone below the diaphragm have elevated basal FSH levels, and 18 of these also have elevated basal LH levels. Median testicular volume is 11 ml (range 5–25 ml). Eleven of 13 patients investigated are azoospermic. All patients have normal testosterone levels, and normal secondary sexual characteristics. There is no biochemical evidence of healing of the damaged germinal epithelium with elevated FSH levels persisting up to 17 years from the end of therapy. These results indicate a high incidence of damage to the germinal epithelium in patients treated with ChlVPP chemotherapy and/or radiotherapy below the diaphragm. Appropriate counselling of these patients with regard to their reproductive capabilities is essential.

With a current 5 year event free survival of 90%, there is increasing interest in the late effects of treatment in patients with Hodgkin's disease. Decreased sitting height due to extended field irradiation including the spine (Wilimas *et al.*, 1980) and abnormalities of thyroid function after neck radiotherapy have been well documented (Shalet *et al.*, 1977). Infertility appears to be almost inevitable in adult males with Hodgkin's disease treated by six or more courses of MOPP or MVPP (mustine, vincristine/vinblastine, procarbazine, prednisolone) (Whitehead *et al.*, 1982*a*). Chemotherapy induced testicular damage in patients treated for Hodgkin's disease in childhood was first reported by Sherins *et al.*, in 1978. Whitehead *et al.* (1982*b*) reported 15 males treated with MOPP for Hodgkin's disease in childhood and concluded that severe testicular damage is common, with azoospermia, but normal pubertal development. More recently Brämswig *et al.* (1990) evaluated testicular function in 75 boys treated for Hodgkin's disease with involved or extended field irradiation and chemotherapy with OPPA (vincristine, prednisone, procarbazine, doxorubicin) or COPP (cyclophosphamide, vincristine, prednisone, procarbazine). Testicular dysfunction was observed in boys treated before as well as during puberty. Abnormal basal FSH/LH levels were found more frequently in patients who had received higher cumulative doses of chemotherapy.

We have studied testicular function in 40 males treated in childhood for Hodgkin's disease with chemotherapy (usually chlorambucil, vincristine, procarbazine, prednisolone – ChlVPP) (Robinson *et al.*, 1984) and/or radiotherapy to assess the effect of this treatment on subsequent fertility.

### Method

Testicular function was evaluated in males treated for Hodgkin's disease in childhood at St Bartholomew's Hospital and the Hospital for Sick Children, London, between 1971 and 1985. All patients included in the study were 16 years or over (median 23 years, range 16 years 8 months–30 years) at the time of their most recent evaluation and had been off treatment for a minimum of 6 years (median 11 years 9 months, range 6–18 years). Basal FSH, LH and testosterone levels were measured. FSH and LH levels were measured by standard immunoradiometric assays with intra- inter-assay coefficient of variation (CV) of $<5\%$ for both assays. Testosterone was measured by radioimmunoassay with a CV of $<5\%$. Testicular size was measured using a Prader orchidometer, and all patients were given the opportunity to have a seminal analysis performed.

For the purpose of this study we defined abnormal testicular function using the following criteria. Germ cell dysfunction was considered present if basal FSH level was raised above $8\ u\ l^{-1}$. Confirmatory evidence for this was a low testicular volume ($<15$ ml, Zachmann *et al.*, 1974), and a low sperm count. Leydig cell dysfunction was considered present if basal LH level was raised above $10\ u\ l^{-1}$ with or without a low testosterone level ($<9\ nmol\ l^{-1}$).

### Clinical details

Seventy-five males were treated for Hodgkin's disease between 1971–1985, 45 at St Bartholomew's Hospital and 30 at the Hospital for Sick Children. Fifteen patients have died, nine have been lost to follow up, six are followed up at other hospitals, and five are still under 16 years of age. Forty patients are over the age of 16 years and have been off treatment for more than 6 years. These patients form the study population. Patient characteristics are shown in Table I.

Of the 28 patients who were treated with chemotherapy, 22 received ChlVPP (median six courses, range 3–8) and five patients treated between 1971 and 1976 had MOPP or MVPP chemotherapy (median six courses, range 3–20). One patient was treated with six courses of COPP. The median dose and range of drugs known to be toxic to the gonads is shown in Table II. Three patients received additional chemotherapy with ABVD (doxorubicin, bleomycin, vinblastine and DTIC), and one of these also had two courses of PAVE (prednisolone, doxorubicin, vinblastine and etoposide) and four courses of CCNU, chlorambucil and etoposide for resistant disease.

Five patients who had chemotherapy also had radiotherapy below the diaphragm (two to para aortic glands, 2,250 and 2,500 cGy; one inverted Y, 2,750 cGy; one coeliac axis, 2,500 cGy; and one whole abdomen after abdominal

Correspondence: E.A. Shafford.
Received 19 May 1993; and in revised form 28 July 1993.

**Table I** Patient characterstics

| | | |
|---|---|---|
| Age at diagnosis | median 10 yrs 5 m'ths (range 4 yrs 3 m'ths–15 yrs 11 m'ths) | |
| Stage at diagnosis | I–14<br>II–13<br>III–12<br>IV–1 | |
| Chemotherapy alone | | 7 |
| Chemotherapy + radiotherapy above diaphragm | | 16 |
| Chemotherapy + radiotherapy below diaphragm | | 1 |
| Chemotherapy + radiotherapy above and below diaphragm | | 4 |
| Total number of patients given chemotherapy | | 28 |
| Radiotherapy above diaphragm | | 7 |
| Radiotherapy below diaphragm | | 4 |
| Radiotherapy above and below diaphragm | | 1 |
| Total number of patients given radiotherapy alone | | 12 |
| Age at evaluation | median 23 yrs (range 16 yrs 8 m'ths–30 yrs) | |
| Median follow up | 12 yrs 6 mn'ths (range 6–20 yrs) | |

**Table II** Median dose $m^{-2}$ of drugs with known gonadal toxicity (range)

| | | |
|---|---|---|
| CCNU | 600 mg $m^{-2}$ | |
| Chlorambucil | 504 mg $m^{-2}$ | (242–760) |
| Cyclophosphamide | 6,310 mg $m^{-2}$ | |
| Mustine | 72 mg $m^{-2}$ | (36–240) |
| Procarbazine | 8,000 mg $m^{-2}$ | (500–33,600) |

**Figure 1** Basal FSH levels pre and post puberty illustrating that damage to the seminiferous epithelium, as indicated by an elevated FSH level, may only become apparent post puberty.

relapse, 3,500 cGy). Twelve patients were treated with radiotherapy alone, seven above the diaphragm, four below the diaphragm, and one above and below the diaphragm.

### Results

### *Chemotherapy*

Twenty-six of 28 patients who had chemotherapy have elevated basal FSH levels, with a median of 18.1 u $l^{-1}$, range 10.1–35.6 u $l^{-1}$ (upper limit of normal 8 u $l^{-1}$). One patient has a normal FSH level after only three courses of ChlVPP at the age of 4 years 10 months and one patient has not had FSH/LH and testosterone levels measured. Eighteen patients have had serial FSH levels measured at least 12 months off treatment and over a minimum of 2 years, and maximum of 13 years (Table III). FSH levels remain elevated for up to 17 years from the end of therapy. In no patient has an elevated FSH level returned to normal. Three patients (1, 4, 8: Table III) had FSH levels measured before or during puberty and more than 1 year off treatment. All had normal levels which subsequently became elevated post puberty (Figure 1).

Sixteen patients also have elevated basal LH levels greater than 10.0 u $l^{-1}$, with a median of 12.3 u $l^{-1}$, range 10.1–24.0 u $l^{-1}$ (upper limit of normal 10 u $l^{-1}$). Eighteen patients have had serial LH measurements (Table III). Four patients (case numbers 4, 7, 12, 15) have always had normal levels. Two patients (2, 18) initially had elevated LH levels 2 years and 4 years 6 months off treatment respectively, which subsequently returned to normal. Four patients (3, 5, 9, 17) have had persistently elevated levels and eight patients (1, 6, 8, 10, 11, 13, 14, 16) all except one of whom (1) were post pubertal, initially had normal LH levels which have subsequently become elevated with increasing time from the end of treatment (Figure 2). Testerone level is normal in all 25 patients in whom it was measured (median 14.5 nmol $l^{-1}$, range 8–30 nmol $l^{-1}$). Whilst all testosterone levels remain in the normal range, three patients have shown post pubertally a sustained fall in testosterone level over 4 years (Table III, 10), 7 years (Table III, 15) and 12 years (Table III, 13) which in two patients (10, 13) has been associated with a rise in previously normal LH levels. Five other patients may be demonstrating a similar trend (Table III, 3, 5, 8, 16, 18).

The median testicular volume for 27 patients is 11 ml (range 5–25 ml). Seventeen patients have testicular volumes of 12 ml or less. So far only 12 patients have had a seminal analysis performed, and 11 are azoospermic after a median of six courses of ChlVPP (range 3–8) and a median of 10 years (range 3 years 6 months–15 years) off treatment. The patient who is infertile after only three courses of ChlVPP was treated at the age of 13 years and did not receive any abdominal radiotherapy (Table III, 13). All of these 11 patients have raised FSH levels, 7/11 also have raised LH levels, and 7/11 have testicular volumes of 12 ml or less. One patient is severely oligospermic with a sperm count of $0.4 \times 10^6$ $ml^{-1}$, and 49% abnormal forms, 4 years following treatment with four courses of ChlVPP and six courses of ABVD. His FSH level is elevated, but LH and testosterone levels and testicular volumes are normal. Nevertheless, this patient has fathered two sons 5 and 6 years off treatment (Table III, 15). Another patient has a baby daughter 11 years after treatment with ChlVPP × 6, but FSH/LH, testosterone levels, and testicular volumes are not available on this patient. However, as Schwartz (1990) pointed out, the reported fathering of a child is not necessarily conclusive proof of fertility. None of the other patients have children.

### *Radiotherapy*

Of 12 patients who were treated with radiotherapy alone, seven had radiotherapy to sites above the diaphragm, and all have normal FSH, LH and testosterone levels, and normal testicular volumes. One patient who requested a seminal analysis has a normal sperm count.

Four patients had radiotherapy alone to sites below the diaphragm and one patient had radiotherapy above and below the diaphragm. Three patients received 3,500 cGy to an inverted Y field, all have elevated FSH levels and two have elevated LH levels. One of these patients initially had a normal FSH level which became elevated during puberty (Figure 1). Testosterone levels are normal, but all patients have small testes – 12 ml or less. One patient has had a seminal analysis and he is severely oligospermic with a sperm

**Table III** Serial FSH and LH levels in 18 patients treated with chemotherapy ± radiotherapy

| *No. and age at diagnosis* | *Dose (mg m$^{-2}$) of gonadal toxic agents* | *Abdominal radiotherapy (cGy)* | *Age at end of treatment* | *Time off treatment* | *FSH level (u l$^{-1}$)* | *LH level (u l$^{-1}$)* | *Testosterone level (nmol l$^{-1}$)* | *Testicular volumes* | *Seminal analysis* |
|---|---|---|---|---|---|---|---|---|---|
| (1) 4 yrs 8 m'ths | Mustine 60 | – | 6 yrs | 4 yrs | 2.3 | 1.4 | | 12 ml 12 ml | Not done |
| | Procarbazine 8400 | | | 5 yrs | 1.6 | 3.5 | | | |
| | | | | 7 yrs | 10.2 | 4.9 | | | |
| | | | | 9 yrs | >25.0 | 10.8 | | | |
| | | | | 15 yrs | 29.1 | 18.2 | 14.5 | | |
| | | | | 16 yrs | 21.1 | 10.5 | 14.1 | | |
| | | | | 17 yrs | 23.5 | 12.8 | 17.0 | | |
| (2) 7 yrs 7 m'ths | Chlorambucil 336 | – | 15 yrs | 2 yrs | 19.5 | 10.4 | 21.0 | 8 ml 8 ml | Azoospermia |
| | Mustine 240 | | | 11 yrs | 28.0 | 8.8 | 16.3 | | 10 yrs off treatment |
| | Procarbazine 33600 | | | 12 yrs | 24.7 | 9.5 | 12.5 | | |
| | | | | 13 yrs | 35.6 | 9.8 | 17.0 | | |
| (3) 8 yrs 7 m'ths | Chlorambucil 588 | – | 9 yrs 3 m'ths | 10 yrs 10 m'ths | 10.3 | 11.1 | | | |
| | Procarbazine 9800 | | | 11 yrs 11 m'ths | 8.5 | 11.1 | 17.6 | 8 ml 10 ml | Not done 13 yrs |
| | | | | 13 yrs 10 m'ths | 10.1 | 10.1 | 13.5 | | |
| (4) 9 yrs 4 m'ths | Chlorambucil 336 | Para | 9 yrs 11 m'ths | 1 yr 3 m'ths | 2.6 | 2.0 | 0.9 | 15 ml 15 ml | Azoospermia |
| | Procarbazine 5600 | aortic | | 11 yrs | 23.6 | 5.1 | 12.0 | | 12 yrs off treatment |
| | | glands | | 12 yrs | 16.6 | 7.1 | | | |
| | | 2,250 | | 13 yrs | 19.8 | 5.4 | | | |
| | | | | 14 yrs | 20.3 | 6.3 | 13.5 | | |
| (5) 9 yrs 4 m'ths | Chlorambucil 468 | – | 10 yrs | 9 yrs | 20.4 | 18.8 | | 15 ml 10 ml | Azoospermic |
| | Procarbazine 7700 | | | 11 yrs | 18.7 | 10.5 | | | 15 yrs off treatment |
| | | | | 14 yrs | 19.2 | 12.6 | 22.0 | | |
| | | | | 15 yrs | 21.7 | 15.4 | 13.5 | | |
| (6) 10 yrs 7 m'ths | Mustine 132 | – | 12 yrs 6 m'ths | 4 yrs | 15.5 | 6.0 | 7.2 | 8 ml 8 ml | Not done |
| | Procarbazine 6300 | | | 4 yrs 6 m'ths | 13.9 | 5.0 | 18.2 | | |
| | | | | 17 yrs | 21.9 | 14.0 | 22.5 | | |
| (7) 10 yrs 5 m'ths | Chlorambucil 670 | – | 11 yrs 3 m'ths | 7 yrs 2 m'ths | 23.0 | 6.3 | | | |
| | Procarbazine 11200 | | | 10 yrs 7 m'ths | 17.3 | 5.6 | 20.5 | 8 ml 10 ml | Azoospermic |
| | | | | 11 yrs 7 m'ths | 18.0 | 5.8 | | | 12 yrs off treatment |
| (8) 10 yrs 9 m'ths | Chlorambucil 504 | – | 11 yrs 5 m'ths | 3 yrs | 3.0 | 4.8 | 0.6 | 15 ml 15 ml | Azoospermic |
| | Procarbazine 8400 | | | 5 yrs 6 m'ths | 14.1 | 5.0 | 22.5 | | 7 yrs off treatment |
| | | | | 6 yrs 6 m'ths | 13.6 | 6.9 | 20.0 | | |
| | | | | 9 yrs 6 m'ths | 17.2 | 6.4 | – | | |
| | | | | 10 yrs | 27.4 | 10.8 | 29.0 | | |
| | | | | 10 yrs 6 m'ths | 14.1 | 7.5 | 17.3 | | |
| | | | | 12 yrs | 16.4 | 11.9 | 14.0 | | |
| (9) 10 yrs 10 m'ths | Chlorambucil 420 | – | 11 yrs 3 m'ths | 4 yrs 10 m'ths | 25.6 | 13.4 | 18.8 | | |
| | Procarbazine 8400 | | | 5 yrs 10 m'ths | 24.9 | 13.1 | | 8 ml 10 ml | Not done |
| | | | | 6 yrs 10 m'ths | 22.8 | 10.6 | 11.0 | | |

**Table III** (continued)

| *No. and age at diagnosis* | *Dose ($mg\,m^{-2}$) of gonadal toxic agents* | *Abdominal radiotherapy (cGy)* | *Age at end of treatment* | *Time off treatment* | *FSH level ($u\,l^{-1}$)* | *LH level ($u\,l^{-1}$)* | *Testosterone level ($nmol\,l^{-1}$)* | *Testicular volumes* | *Seminal analysis* |
|---|---|---|---|---|---|---|---|---|---|
| (10) 11 yrs 3 m'ths | Chlorambucil 504 | Whole | 16 yrs 8 m'ths | 1 yr 6 m'ths | 23.5 | 8.4 | | 8 ml 8 ml | Azoospermia |
| | Procarbazine 8400 | abdomen | | 3 yrs 6 m'ths | 22.0 | 7.5 | 16.0 | | 3.5 yrs off treatment |
| 16 yrs 3 m'ths | for abdominal relapse | 3,500 | | 4 yrs | 32.7 | 13.0 | 21.3 | | |
| at relapse | | | | 7 yrs | 23.0 | 12.0 | 19.0 | | |
| | | | | 8 yrs | 25.3 | 18.5 | 9.6 | | |
| (11) 11 yrs 4 m'ths | Chlorambucil 552 | – | 12 yrs | 4 yrs | 19.7 | 6.6 | 17.1 | 10 ml 8 ml | Not done |
| | Procarbazine 9100 | | | 8 yrs | 17.5 | 12.7 | 13.5 | | |
| | | | | 10 yrs | 13.8 | 12.1 | 18.0 | | |
| (12) 12 yrs 9 m'ths | Chlorambucil 588 | – | 13 yrs 4 m'ths | 2 yrs | 14.3 | 4.2 | 12.5 | 15 ml 15 ml | Not done |
| | Procarbazine 9800 | | | 3 yrs | 19.5 | 7.6 | 20.1 | | |
| | | | | 6 yrs | 12.9 | 8.5 | 20.1 | | |
| (13) 13 yrs | Chlorambucil 252 | – | 13 yrs 5 m'ths | 2 yrs | 17.5 | 2.9 | 31.9 | 12 ml 12 ml | Azoospermia |
| | Procarbazine 4200 | | | 6 yrs | 19.6 | 7.4 | – | | 5 yrs and 10 yrs |
| | | | | 10 yrs | 18.1 | 7.3 | – | | off treatment |
| | | | | 11 yrs | 27.5 | 11.6 | 16.0 | | |
| | | | | 14 yrs | 14.8 | 10.2 | 10.0 | | |
| (14) 14 yrs 2 m'ths | Chlorambucul 840 | – | 15 yrs 9 m'ths | 2 yrs 6 m'ths | 10.0 | 6.9 | 15.6 | 12 ml 10 ml | Azoospermia |
| | Procarbazine 8400 | | | 4 yrs | 9.9 | 8.2 | – | | 8 yrs off treatment |
| | DTIC 700 | | | 8 yrs | 19.5 | 9.5 | 25.5 | | |
| | CCNU600 | | | 10 yrs | 18.4 | 14.9 | 20.5 | | |
| (15) 14 yrs 4 m'ths | Chlorambucil 336 | – | 15 yrs 4 m'ths | 4 yrs | 22.6 | 8.0 | 16.4 | 20 ml 15 ml | Oligospermic |
| | Procarbazine 5600 | | | 8 yrs | >50.0 | 4.7 | 13.5 | | $0.4 \times 10^6\,ml^{-1}$ |
| | | | | 11 yrs | 11.2 | 7.9 | 10.0 | | 49% abnormal |
| | | | | | | | | | 25% good motility |
| | | | | | | | | | 4 yrs off treatment |
| (16) 14 yrs 9 m'ths | Chlorambucil 504 | – | 15 yrs 4 m'ths | 8 yrs | 19.1 | 7.6 | 16.9 | 12 ml 12 ml | Azoospermia |
| | Procarbazine 8400 | | | 8 yrs 6 m'ths | 25.1 | 11.9 | 20.1 | | 8 yrs off treatment |
| | | | | 10 yrs | 15.0 | 12.6 | 15.5 | | |
| | | | | 11 yrs | 15.9 | 11.4 | 12.5 | | |
| (17) 14 yrs 9 m'ths | Chlorambucil 504 | – | 15 yrs 4 m'ths | 2 yrs 4 m'ths | 15.5 | 10.3 | | 6 ml 6 ml | Azoospermia |
| | Procarbazine 8400 | | | 5 yrs | 14.6 | 17.0 | | | 10 yrs off treatment |
| | | | | 8 yrs | 17.3 | 10.1 | | | |
| | | | | 9 yrs | 16.9 | 14.3 | 14.9 | | |
| | | | | 10 yrs 4 m'ths | 20.4 | 16.2 | | | |
| (18) 15 yrs 11 m'ths | Chlorambucil 588 | – | 16 yrs 7 m'ths | 4 yrs 6 m'ths | 17.4 | 13.3 | | 12 ml 15 ml | Not done |
| | Procarbazine 500 | | | 6 yrs | 9.6 | 6.7 | 18.6 | | |
| | | | | 7 yrs | 11.8 | 4.5 | 14.5 | | |

**Figure 2** Rise in basal LH levels with increasing time from the end of therapy.

count of $0.4 \times 10^6$ ml$^{-1}$, 16 years off treatment. Two patients received 3,500 cGy to the right groin. Both have normal FSH, LH and testosterone levels, but one has small testes (8 ml, 10 ml). For nine of the total of ten patients who received radiotherapy below the diaphragm (five patients treated by radiotherapy alone and five treated by a combination of radiotherapy and chemotherapy), it is not possible to estimate the dose to the testes which are out of the primary beam and further protected by a scrotal lead shield. However, one patient treated with radiotherapy alone, 3,500 cGy to an inverted Y field with a further 500 cGy to the right inguinal region, had a measured total scrotal dose of 456 cGy in 20 fractions over 28 days. At that time he had bilaterally undescended testes and subsequently had a right orchidopexy. Testicular volumes are 2 ml right and 10 ml left.

### Discussion

A number of previous studies have reported on reproductive function following treatment for Hodgkin's disease in childhood with MOPP (Whitehead *et al.*, 1982*b*; Ortin *et al.*, 1990) and OPPA/COPP (Brämswig *et al.*, 1990).

We have reported here 40 males treated for Hodgkin's disease, 28 of whom received combination chemotherapy, mostly ChlVPP. Of these 28 patients, 26 (93%) have elevated basal FSH levels, indicating damage to seminiferous tubules. Seventeen of 27 patients have testicular volumes 12 ml or less, indicating reduced testicular size, normal adult testicular volume being equal to or greater than 15 ml (Zachmann *et al.*, 1974). Eleven of 12 patients who have had a seminal analysis are azoospermic, the other patient being oligospermic. This indicates a high incidence of damage to the germinal epithelium in patients treated with this regimen, both before or during puberty. The only patient who does not appear to have any impairment of gonadal function received only three courses of ChlVPP at the age of 4 years 10 months.

The damage caused to the gonad is not just a function of the chemotherapy received. Three of five patients treated with radiotherapy alone, below the diaphragm, have evidence of impairment of gonadal function as shown by elevated FSH levels in three associated with elevated LH levels in two patients. All three have small testes and one who has had a seminal analysis is oligospermic, 16 years off treatment. There is no evidence of recovery of function up to 17 years from diagnosis. This is similar to the Stanford experience (Ortin *et al.*, 1990).

All patients treated with chemotherapy and/or radiotherapy below the diaphragm having been advised of the possibility of infertility were given the opportunity to have a seminal analysis. However, only 13 patients so far have undergone this investigation. The remainder do not wish to know their fertility status at the present time. The desire for information was not related to the age of the patient as the median age of the two groups is the same.

All patients with azoospermia or oligospermia have raised FSH levels, confirming the close correlation between raised FSH levels and germ cell damage (Brämswig *et al.*, 1990; Siimes & Rautonen, 1990).

This study includes 18 patients who have had serial FSH and LH measurements. It has been suggested that serial FSH levels might help to determine whether the damage sustained by the germinal cell epithelium is in the process of healing (FSH decreasing), stable, or progressive (FSH increasing) (Schwartz, 1990). If basal FSH truly reflects damage to the germinal cell epithelium, then the results shown in Table III do not give grounds for optimism regarding healing of chemotherapy induced damage, with elevated levels persisting up to 17 years from cessation of treatment, suggesting that the damage to the germinal epithelium is irreversible. Figure 1 demonstrates graphically that FSH levels are unhelpful in predicting testicular damage in pre pubertal and peripubertal boys, confirming the findings of Green *et al.* (1981).

All the patients in the study progressed through puberty satisfactorily with the normal development of secondary sexual characteristics which would indicate normal Leydig cell function at that time. None of the patients had gynaecomastia unlike the boys in Sherins study (Sherins *et al.*, 1978). However, 16/28 treated with chemotherapy and 2/5 treated with radiotherapy alone (inverted Y 3,500 cGy) have elevated serum LH levels, first noted 5–19 years from diagnosis. As all testosterone levels are in the normal range, this suggests that increased LH secretion is necessary to maintain normal testosterone production. However with the fall in testosterone levels noted in several patients, premature Leydig cell failure is a real possibility. Continuing follow up of these patients with annual measurement of FSH/LH and testosterone levels is needed to further elucidate the natural history of the impairment of gonadal function.

Appropriate counselling of boys treated with ChlVPP chemotherapy and/or radiotherapy below the diaphragm with regard to their reproductive potential is essential. However, it is important to remember that FSH levels in pre- and peri-pubertal boys are unreliable as indicators of gonadal damage. Although FSH level and testicular size in patients who are post pubertal may give a good indication of damage to the germinal epithelium, seminal analysis still remains the definitive test of an individual's reproductive potential.

Annual follow up, for many years, will be needed before the consequences of ChlVPP-induced damage are fully revealed. There is an urgent need for chemotherapy regimens effective in Hodgkin's disease which are less damaging to the gonads.

### References

BRÄMSWIG, J.H., HEIMES, U., HEIERMANN, E., SCHLEGEL, W., NIESCHLAG, E. & SCHELLONG, G. (1990). The effects of different cumulative doses of chemotherapy on testicular function. *Cancer*, **65**, 1298–1302.

GREEN, D.H., BRECHER, M.L., LINDSAY, A.N., YAKAR, D., VOORHESS, M.L., MACGILLIVRAY, M.H. & FREEMAN, A.I. (1981). Gonadal function in paediatric patients following treatment for Hodgkin's Disease. *Med. Ped. Onc.*, **9**, 235–244.

ORTIN, T.T., SHOSTAK, C.A. & DONALDSON, S.S. (1990). Gonadal status and reproductive function following treatment for Hodgkin's disease in childhood: the Stanford experience. *Int. J. Radiat. Onc. Biol. Phys.*, **19**, 873–880.

ROBINSON, B., KINGSTON, J., NOGUEIA COSTA, R., MALPAS, J.S., BARRETT, A. & MCELWAIN, T.J. (1984). Chemotherapy and irradiation in childhood Hodgkin's disease. *Arch. Dis. Child.*, **59**. 1162–1167.

SCHWARTZ, C.L. (1990). Creating life on the plateau: reproductive potential in survivors of childhood Hodgkin's disease. *Int. J. Radiat. Onc. Biol. Phys.*, **19**, 1099–1100.

SHALET, S.M., ROSENSTOCK, J.D., BEARDWELL, C.G., PEARSON, D. & JONES, P.H. (1977). Thyroid function following external irradiation to the neck for Hodgkin's disease in childhood. *Clin. Radiol.*, **28**, 511–515.

SHERINS, R.J., OLWENY, C.L.M. & ZIEGLER, J.L. (1978). Gynaecomastia and gonadal dysfunction in adolescent boys treated with combination chemotherapy for Hodgkin's disease. *N. Engl. J. Med.*, **299**, 12–16.

SIIMES, M.A. & RAUTONEN, J. (1990). Small testicles with impaired production of sperm in adult male survivors of childhood malignancies. *Cancer*, **65**, 1303–1306.

WHITEHEAD, E., SHALET, S.M., BLACKLEDGE, G., TODD, I., CROWTHER, D.C. & BEARDWELL, C.G. (1982*a*). The effects of Hodgkin's disease and combination chemotherapy on gonadal function in the adult male. *Cancer*, **49**, 418–422.

WHITEHEAD, E., SHALET, S.M., MORRIS JONES, P.H., BEARDWELL, C.G. & DEAKIN, D.P. (1982*b*). Gonadal function after combination chemotherapy for Hodgkin's disease in childhood. *Arch. Dis. Child.*, **47**, 287–291.

WILIMAS, J., THOMPSON, E. & SMITH, K.L. (1980). Long term results of treatment of children and adolscents with Hodgkin's disease. *Cancer*, **46**, 2123–2125.

ZACHMANN, M., PRADER, A., KIND, H.P., HÄFLIGER, H. & BUDLIGER, H. (1974). Testicular volume during adolescence. Cross-sectional and longitudinal studies. *Helv. Paediatr. Acta*, **29**, 61–72.